# Bioengineering of crop plants for improved tetrahydrofolate production

**DOI:** 10.1080/21655979.2017.1373537

**Published:** 2017-09-21

**Authors:** Bhupendra Chaudhary, Nagendra Singh, Dhananjay K. Pandey

**Affiliations:** School of Biotechnology, Gautam Buddha University, Greater Noida, U.P. India

**Keywords:** allosteric regulation, folates, phylogeny, plant bioengineering

## Abstract

*De novo* synthesis of folates in plants is tightly regulated through feedback-regulation of certain pathway catalysts. Recently, we investigated the prospects of incessant production of folates in an evolutionary conjunction, through the overexpression of feedback targeted and evolutionarily conserved heterologous *E.coli* dihydroneopterin aldolase (*Ec*DHNA) in tobacco.^1^ The enhanced production of folates in the transgenic lines was associated with differential allosteric regulatory cavities accessible at *Ec*DHNA surface having critical amino-acid differences as Ile 64 (His_63), Val 70 (Phe_69), His 75 (Arg_78) and Arg 79 (Glu_72). These structural characteristics are indicative of evolutionary signatures of the catalytic feedback-regulation of folate manufacturing. We exploited the biotechnological potential of such allosterically diverged *trans*-DHNA for improved folate production in plants. Nonetheless, genetic manipulation of single enzymes modulating complex pathways such as folate biosynthesis is often inadequate to achieve desired phenotypes; therefore, multi-gene integration with explicit genic-combination for folate enrichment in plants has also been projected for future folate agri-biofortification schemes.

## Introduction

**Figure 1.**
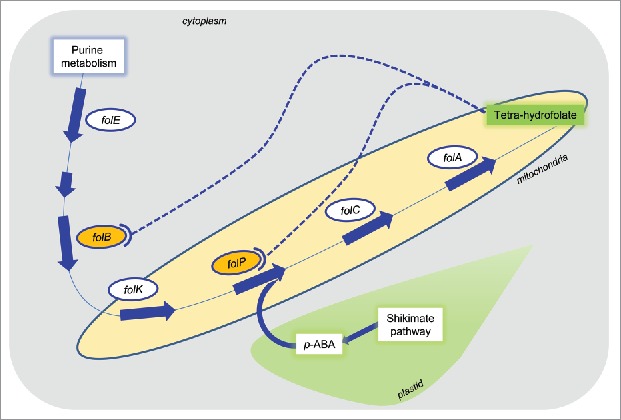
Folic acid biosynthetic pathway in plants illustrating key catalysts involved at different steps. These catalysts are primarily required for the production of pterins and p-ABA in the plant cell. The pterins are produced in the cytoplasm by purine metabolism and carried to mitochondria, whereas the p-ABA branch is initiated in plastids through shikimate pathway and subsequently p-ABA is transported to mitochondria for the production of folic acid in association with pterins. Certain folic acid rate-determining catalysts such as DHNA (folB) and DHPS (folP) are known for the regulation of folic acid production in the cell mainly through potential feedback regulation.

Vitamin B_9_ (folic acid or folate) in combination with other vitamins contributes in the neural tube development of the human fetus and prevent other birth defects, such as cleft palate and heart abnormalities.^2^ *De novo* synthesis of folates usually occurs in plants and microorganisms whereas humans need dietary folates preferably from plant sources such as green vegetables.^3^ In plants, folates are synthesized through multistep pathways in presence of several essential catalyzing proteins. Synthesis of folates is instigated in cytoplasm through the production of pterins and gets completed in mitochondria, whereas *p-*ABA branch is synthesized from chorismate in plastids and transported to mitochondria.^4^ However, the production of folic acid in a plant cell is tightly controlled by the feedback and feedforward regulation involved in the biosynthetic pathway at rate-determining steps (Fig. 1).^4,5^ Therefore, a promising solution to this challenge is to bioengineer the crop plants by the over-expression of evolutionarily diverged key folate biosynthetic enzymes having differential allosteric sites, which may preclude the feedback regulation.

In a plant cell, the expression of DHNA (dihydroneopterin aldolase; EC 4.1.2.25) which catalyzes the conversion of dihydroneopterin into 6-hydroxymethyl-dihydropterin, and *DHPS* (dihydropteroate synthase; EC 2.5.1.15) which catalyses the conversion of 6-hydroxymethyl-dihydropterin into dihydropteroate in presence of *p-*ABA are majorly subjected to strong feedback regulation.^6^ The DHNA sequence comprises of different motifs among bacterial species having hyper-variable regions adjoining to the catalytic sites.^7^ Biochemical analysis of DHNAs from *E. coli* and *S. aureus* showed dissimilarities in their ligand binding sites and catalytic properties.^8^ Thex-ray crystallography of DHNA proteins has performed for their structural determination across bacterial and plant species such as *S. aureus*,^9^ *M. tuberculosis*,^10^ *S. pneumoniae*,^11^ and also in *Arabidopsis thaliana.**^12^* These crystal structures have thus facilitated the characterization of their catalytic mechanisms. Further, molecule dynamics simulation analysis of substrate complex has revealed the structural basis of these biochemical differences that attributed to varied catalytic mechanisms. Such structural variations in the near vicinity of ligand binding sites underlie substitution of amino acid residues. This complex-simulation had precisely revealed the presence of persistent and transient hydrogen bonding between substrate and enzyme complex.^7^

Recently, we reported the enhanced production of folates in tobacco leaves by the constitutive overexpression of recombinant *DHNA* gene of *E.coli* (*Ec*DHNA) having differential allosteric product-binding sites at its surface.^1^ Our approach was to identify novel allosteric sites at the *Ec*DHNA protein surface as compared with *Arabidopsis* DHNA (*At*DHNA), and utilize the allosterically diverged *Ec*DHNA gene for the genetic transformation of tobacco plants to improve the folic acid production. Also, the overexpressed bacterial DHNA protein was significantly correlated with the intracellular folate production in *E. coli* cells, assuming its potential usage for crop biofortification. Here, we provide additional data on the evolutionary characterization and homologies based allosteric site prediction at the surface of DHNA protein of bacterial and plant origins for the designing of genome engineering strategies, and also highlight the need of gene pyramiding for future crop biofortification programs.

### Orthologous DHNA diversity and their evolutionary relatedness

**Figure 2.**
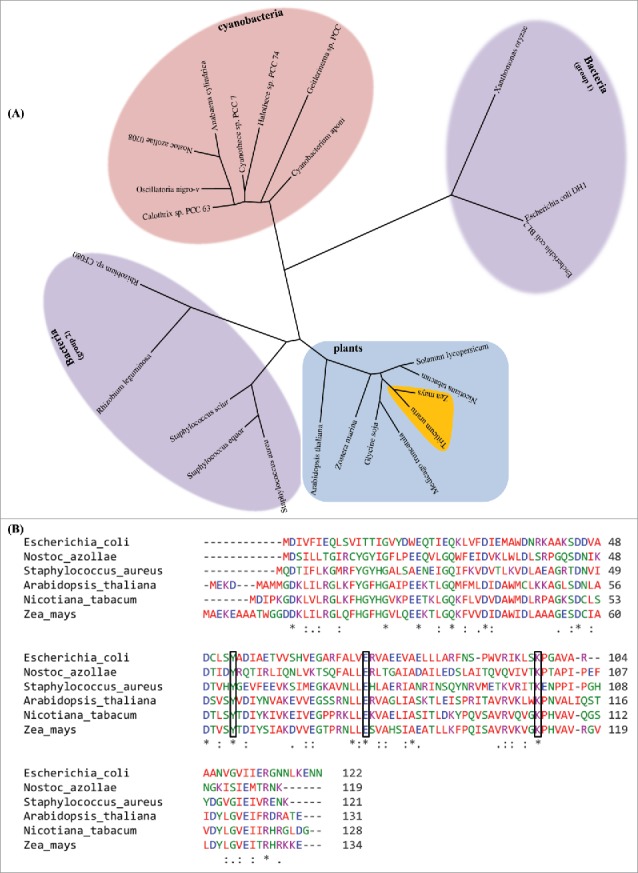
Phylogenetic analysis of DHNA protein sequences of representative bacterial, cyanobacterial and plant species. (A) Unrooted phylogram of DHNA protein sequences of representative bacterial, cyanobacterial and plant species developed using NJ method. The sequences are deduced from KEGG database. The protein sequences are aligned using Phylogeny.fr online tool. Different colour backgrounds highlight distinct clades of plants (both monocots and dicots), bacteria and cyanobacteria. Bacterial representatives exhibited two distinct clades showing more proximity to plant or cyanobacterial species than other bacteria, indicating towards the horizontal evolution of DHNA protein across taxa. In plant clade, monocot plants (highlighted with colored triangle) are aligned with other dicot plant representatives and indicate for the paraphyletic evolution of DHNA protein among plant species. (B) DHNA protein sequence alignment of six diverse species representing bacterial, cyanobacterial and plant (monocot and dicot) representatives. At least thirteen highly conserved amino acid residues were identified and marked with asterisks. Selective conserved residues-Lys98, Try53 and Glu73 (highlighted with red colored box) were considered to be responsible for the catalytic site architecture, and have been previously shown with SDM experiments.

To analyze the diversity and evolutionary correlation among DHNA proteins in the major class of folic acid producing species *i.e.* bacteria, cyanobacteria and plants, the amino acid sequences were aligned using “*A la Carte*” mode of Phylogeny.fr online tool. In result, an unrooted phylogram was constructed having distinct clades of plants (both monocots and dicots), bacteria and cyanobacteria. Among bacterial DHNA sequences, *E. coli* and *Xanthomans* shared maximum sequence similarity whereas nodulating bacteria *Rhizobium* sp. showed prominent evolutionary relatedness with cyanobacteria members. On the contrary, selective bacterial representatives exhibited relatively more proximity to plants or algal species than other bacteria (Fig. 2A). Based on phylogenetic analyses of prevalent species representatives, evolution of DHNA is suggestive to be paraphyletic in nature. Interestingly, plant phylogenetic group consisting of both monocot and dicot members illustrated considerable resemblances in their protein coding sequences, and highlight for their conserved role in folate biosynthesis. However, slight amino acid variations between plant and bacterial DHNA sequences may have attributed to the existing diversity in protein folding and ultimately in the active and allosteric site architecture at the protein surface. Therefore, multiple sequence alignment was performed to identify the essential amino acids responsible for the catalytic site architecture and their degree of conservation across species. The sequence alignment identified at least 13 conserved amino acids amongst prevalent species representatives (Fig. 2B). All of these, three amino acid residues- Lys98, Try53 and Glu73 in *Ec*DHNA have been previously experimented for their direct involvement in the formation of catalytic site responsible for the binding with neopterin substrate (Fig. 2B).^13^ Further, we identified a thoroughly conserved Try53 residue which along with Lys98 in *Ec*DHNA constitutes a proton wire for the catalytic site architecture.^14^ Though a handful conserved residues have already been characterized, other conserved residues as identified in the present study may also influence the formation of DHNA catalytic site and are subjected to experimental validation.^7,9,11,13^

The phylogenetic analysis of DHNA orthologs among bacterial, cyanobacterial and plants (monocot and dicot species) revealed certain degree of protein sequence conservation highlighting their functional conservation at evolutionary scale (Fig. 2A). As follows, what are the engineering prospects for transducing a functional analog or heterologous expression of a structurally divergent homolog of a rate-limiting enzyme that may help avoid such metabolic constraint? Previously, a study by Hossain *et al.* (2004) reported that ectopic overexpression of *folA* enzyme in folate biosynthetic pathway led to very high production of pterins in the cell, whereas little increase in the folate level was observed. Therefore, important enzymes such as DHNA and DHPS that are subjected to feedback regulation may be genetically manipulated in crop plants by the overexpression of heterologous proteins having divergent protein-regulation sites.

### Prediction of allosteric regulatory sites at the surface of DHNA catalyst

**Figure 3.**
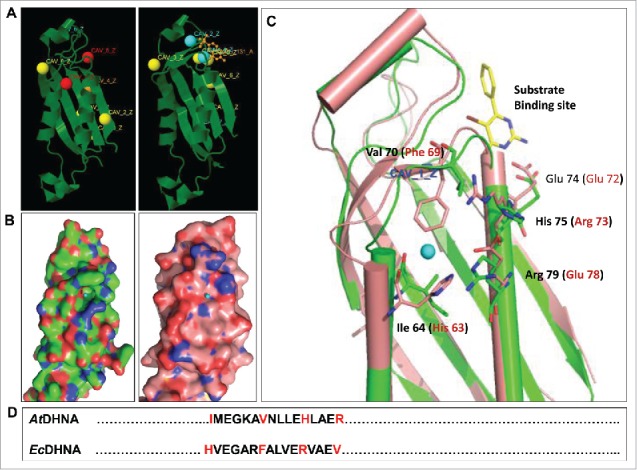
(A) PARS based comparative localization of allosteric regulatory sites on the surfaces of AtDHNA and EcDHNA (most significant predicted sites are shown in red; least significant predicted sites are shown in yellow/cyan). (B) The panel highlights the molecular surface representations for the allosteric sites (marked in blue) in the AtDHNA (green) and EcDHNA (pink) protein structures. (C) Superimposition of the crystal structures of AtDHNA (green) and EcDHNA (pink) proteins, showing difference in the predicted allosteric sites. (D) Amino acid sequence comparison of the predicted allosteric site (64-79) on AtDHNA (PDB ID:1SQL) with the corresponding region (64-79) of EcDHNA (PDB ID:2O90).

To determine if the ectopic expression of a heterologous *Ec*DHNA protein in plants would lead to the independent and uninterrupted synthesis of tetrahydrofolate, *in-silico* predictions of allosteric regulatory sites at *Ec*DHNA and *At*DHNA surfaces were predicted using PARS, a server for the prediction of allosteric and regulatory sites on protein structures.^14^ The PARS server assesses the alterations in protein dynamicity and structural conservation to identify pockets that may exert a regulatory effect upon binding of a small-molecule ligand. This server helps predict the single allosteric point within 10 Å of an allosteric modulator crystal structure using normal modes which is more significant than the Fpocket algorithms to predict allosteric sites by other prediction tolls such as AlloSite or AlloPred.^14^ The protein coordinates of *Ec*DHNA and *At*DHNA were extracted from the Protein Data Bank (PDB) with accession codes 2O90 and 1SQL, respectively. *In silico* prediction of allosteric sites in both *Ec*DHNA and *At*DHNA responsible for protein-regulation mechanism was performed. This prediction analysis has identified seven and eight surface allosteric regulatory cavities on *Ec*DHNA and *At*DHNA, respectively. One common cavity (CAV_5_Z) in both the proteins was identified as substrate binding site (Fig. 3A). Six sites in *Ec*DHNA and seven sites in *At*DHNA were found to be non-significant in terms of their confidence scores. Among other identified sites, the most significant site was observed as CAV_1_Z which is exclusively present at *At*DHNA surface (Fig. 3B). The site CAV_1_Z is located in the close proximity to the active site with a good confidence score and considered important for the cellular folate turnover through feedback regulation. Structural comparison revealed at least four amino acid mutations in both the enzymes. The critical differences of amino acids were observed as Ile 64 (His_63), Val 70 (Phe_69), His 75 (Arg_78) and Arg 79 (Glu_72) in the allosteric sites of both the enzymes (Fig. 3C, D). These mutations cumulatively may have greatest impact on the enzyme's allosteric binding behavior for tetrahydrofolate in the cell. The amino acid alterations have created a large difference in the charge distribution at the allosteric surface of both the enzymes. Additionally, presence of Phe residues along with its conformation in *Ec*DHNA has physically blocked the cavity, which rendered no place for binding of allosteric regulator to *Ec*DHNA enzyme (Fig. 3B). These differences of amino acids provide the structural basis of presence of a well-defined allosteric site on *At*DHNA and a flat surface of the *Ec*DHNA protein, hence the exclusive allosteric regulation of *At*DHNA by cellular folates (Fig. 3B). This may result in uninterrupted catalytic activity of these proteins even at high concentration of folates, thus circumventing the feedback regulation. Thus bacterial DHNA may serve as a heterologous protein in the crop plants growing in widespread agri-climates, as characterized previously for its highly stable activity at varied temperature conditions.^1^

### Overexpression of heterologous *Ec*DHNA enhanced THF production in transgenic tobacco

The bifunctional *Ec*DHNA gene is 369 nucleotide long nucleotide sequence encoding 122 aa peptide chain and a protein of ∼13.6kDa. Recently, we have shown that *Ec*DHNA gene was successfully overexpressed in the induced M-15 bacterial host cells and the temporal estimation of extracellular and intracellular folic acid was analyzed.^1^ Remarkably, the maximum concentration of intracellular folic acid concentration was observed at 4 hr of growth under induced condition in the recombinant DHNA samples as compared with the control samples. Nevertheless, a significant decline in the folic acid content was measured at 6 hr growth time-interval, indicating for the inception of stationary phase in all induced culture samples thus adversely affecting the cellular growth and metabolism.

Could the folate levels be increased by ectopic over-expression of candidate folate biosynthetic pathway genes in plants? Based on the significant correlation between heterologous overexpression of DHNA gene and folate over-production, we assumed that the bacterial *Ec*DHNA protein having divergent allosteric sites may potentially be utilized for crop biofortification. In this direction, *Agrobacterium*-mediated genetic transformation of tobacco was performed and the transformed leaf explants could be regenerated following the process of organogenesis *in vitro*. Constitutive overexpression of *Ec*DHNA gene was achieved in the transgenic plants using a viral CaMV35S promoter and *neomycin phosphotransferase* II (*npt*II) gene cassette as plant selection marker.^1^ The folate content was measured in the growing buds of independent transgenic lines using HPLC. In result, more than twofold enrichment for the folate levels in the transformed tissues was evident as compared with WT. These results are remarkable as they highlight the contribution of structurally diverged bacterial *Ec*DHNA to the cellular folate production under crucial metabolic constraints in cellular folate biosynthesis mainly through tight feedback regulation of native aldolases. The use of bacterial DHNA gene has definitely persuaded the turnover of end product in folate biosynthetic pathway that may have incredible impact on crop nutrigenomics programs.

### Need of gene pyramiding for folate biofortification in crop plants

Low amounts of folates in crop plants may be largely attributed to the tight feedback regulation of certain folate biosynthetic enzymes. Up to now, various plant species have been genetically modified with individual folate biosynthetic pathway genes for folate enhancement,^6,15,16^ however, very low increment in the cellular folate concentration was recorded mainly due to very tight regulation of folate production in the cell.^17^ To counter the problem of low vitamin production in plants, ‘multi-gene integration for single trait’ approach for folate enrichment is necessary but a genic-combinatorial view of folate vitamin enhancement in plants through genetic approaches is yet to be explored. The overexpression of *folE* gene enhanced pterins biosynthesis up to 1250-fold without necessarily allaying with folate production in transgenic *Arabidopsis*,^6^ tomato^15^ and corn.^18^ Unexpectedly, pyramiding of *folE* enzyme with aminodeoxychorismate synthase (*ADCS*) in *p*-ABA branch resulted into inadequate variation of the folate concentration in plants.^16,19^ These enzymes are essential at initial steps of folate biosynthesis whereas successive rate-determining enzymatic steps are tightly regulated by potential feedback mechanism, thus controlling the folate turnover in the cell. Therefore, arbitrary pyramiding of folate biosynthetic genes overproducing pterins and *p*-ABA branch enzymes may not suffice for the ultimate genic-combination of folate vitamin enhancement in plants. If so, what genic-combination(s) should be preferred for improved folate production in the cell?

Evidently, an aldolase enzyme DHNA (*folB*) responsible for the formation of 6-hydroxymethyl-dihydropterin in folate biosynthesis pathway and DHPS (*folKP*) responsible for the formation of dihydropteroate are subjected to feedback regulation (Fig. 1). Recently, we have shown that overexpression of bacterial *Ec*DHNA gene potentially regulates folate biofortification in tobacco.^1^ Since *Ec*DHNA has evolved with divergent allosteric sites at its surface which may avoid folate feedback regulation, it's pyramiding with other candidate genes of folate biosynthetic pathway such as *folE* and *ADCS* would be a promising experimental combination of genes for future folate metabolic engineering. As performed in our study,^1^ an alternate strategy would be to develop separate transgenic stocks of the direct targets of folate feedback regulation *i.e. DHNA* and *DHPS* genes and investigate their discrete impact on the folate production. Also, pyramiding of these two transgenes in the field through cross-pollination and analyze their collaborative impact on the folate production in F_1_ generation would be of tremendous scientific interest providing evidences for the genetics of folate agri-biofortification.
